# Multivariate multilevel spline models for parallel growth processes: application to weight and mean arterial pressure in pregnancy

**DOI:** 10.1002/sim.5385

**Published:** 2012-06-26

**Authors:** Corrie Macdonald-Wallis, Debbie A Lawlor, Tom Palmer, Kate Tilling

**Affiliations:** a MRC Centre for Causal Analyses in Translational Epidemiology, School of Social and Community Medicine, University of BristolU.K; b School of Social and Community Medicine, University of BristolU.K

**Keywords:** linear spline, multivariate, multilevel model, structural equation model, longitudinal, change

## Abstract

Growth models are commonly used in life course epidemiology to describe growth trajectories and their determinants or to relate particular patterns of change to later health outcomes. However, methods to analyse relationships between two or more change processes occurring in parallel, in particular to assess evidence for causal influences of change in one variable on subsequent changes in another, are less developed. We discuss linear spline multilevel models with a multivariate response and show how these can be used to relate rates of change in a particular time period in one variable to later rates of change in another variable by using the variances and covariances of individual-level random effects for each of the splines. We describe how regression coefficients can be calculated for these associations and how these can be adjusted for other parameters such as random effect variables relating to baseline values or rates of change in earlier time periods, and compare different methods for calculating the standard errors of these regression coefficients. We also show that these models can equivalently be fitted in the structural equation modelling framework and apply each method to weight and mean arterial pressure changes during pregnancy, obtaining similar results for multilevel and structural equation models. This method improves on the multivariate linear growth models, which have been used previously to model parallel processes because it enables nonlinear patterns of change to be modelled and the temporal sequence of multivariate changes to be determined, with adjustment for change in earlier time periods. Copyright © 2012 John Wiley & Sons, Ltd.

## 1 Introduction

Growth models are often used in life course epidemiology to describe patterns of growth or change, to assess the influences of rates and timing of change on later health outcomes and to investigate the effects of exposures on patterns of change [1–7]. In some cases two or more change processes occur in parallel, such as gains in height and weight during childhood, and it may be of interest to assess relationships between such processes, or to adjust for the effects of one change process when assessing the associations of another with a later health outcome. It may also be hypothesised that change in one process causes a change in another, or that an unmeasured or latent construct causes change in both of the processes simultaneously. Bivariate multilevel models (MLMs) for repeated measurements with linear relationships of both outcome variables with time have been used to demonstrate the correlation between values of the two variables at baseline and rates of change in the variables over time [7–9]. However, this does not demonstrate whether change in one variable temporally precedes change in another and is thus unable to provide evidence towards a causal effect.

Linear spline models have recently been proposed as a method of representing growth 2012 or change 2010, which reduces the dimensionality of repeated measurements. The shape of the trajectory of change is assumed to be piecewise linear, with knot points defining changes in the magnitude or direction of association of the response variable with time. The selection of the number and location of knot points may be determined by the data or by prior knowledge and therefore the linear splines represent the shape of the change trajectory in a meaningful way, and the linear periods of change provide coefficients that are more interpretable than for polynomial curves. By first fitting splines to each change process and then modelling these processes in parallel, we are able to model associations between observed rates of change in one variable in one period of time and observed rates of change in another variable in a subsequent period of time, with time periods defined by the splines, and thus we may identify whether changes in one variable precede changes in another. By examining the temporal sequence of events there is the possibility of improving causal inferences for pathways between variables. Furthermore, prior knowledge or hypotheses about which pathways exist may be tested by constraining covariances between individual-level random effects for different periods of change to be zero and comparing the model fit to models where these covariances are freely estimated.

We will use the example of weight and mean arterial pressure (MAP) during pregnancy to demonstrate these models. In normal pregnancy there is a decrease in blood pressure in early pregnancy followed by a rise in late pregnancy [11]. Hypertensive disorders of pregnancy (HDP), defined by high blood pressure in late pregnancy (after 20 weeks' gestation), are associated with risk of adverse health outcomes for both the mother and offspring 12–14. and gestational weight gain (GWG) has been found to be positively associated with the risk of developing an HDP 15–17. However, it is not clear whether changes in weight during pregnancy precede changes in blood pressure, or whether increases in blood pressure precede GWG because of increases in oedema and plasma volume expansion 2009. MAP is the average pressure in an artery over a complete cycle of one heart beat. It is estimated by combining systolic and diastolic blood pressure and allowing for the lower pressure during the diastolic phase of the cardiac cycle (see below for description of how this is calculated). In this paper we have used MAP so that we have one blood pressure variable that we can relate to weight change in pregnancy and that takes account of blood pressure in both systole and diastole. We show how MLMs and structural equation models (SEMs) with linear splines for gestational age can be applied to weight and MAP to learn whether an increase in weight precedes a rise in MAP, and in which periods of pregnancy associations are strongest. The paper is structured as follows: in Section 2 we describe the data. In Section 3 we define the multilevel multivariate response model and the derivation of regression coefficients from the variances and covariances of random effects. In Section 4 we show that the model can be equivalently defined in SEM form. In Section 5 we provide an application of the model to weight and MAP changes in pregnancy, and compare results from fitting the model in MLM software (MLWIN) (Rasbash J, Browne W, Healy M, Cameron B, Charlton C, Centre for Multilevel Modelling, University of Bristol, UK) and SEM software (MPLUS) (Muthen and Muthen, Los Angeles, California). Section 6 is a discussion of the methods described.

## 2 Data

The data are from the Avon Longitudinal Study of Parents and Children (ALSPAC), which is described in full elsewhere 2001 and on the website http://www.bris.ac.uk/alspac. A total of 14,541 pregnant women were recruited, who were living in a defined area of Avon including the city of Bristol during their pregnancy and had an expected delivery date between 1 April 1991 and 31 December 1992. Ethical approval for the study was obtained from the ALSPAC Law and Ethics Committee and from the National Health Service local ethics committee. We restrict analysis to singleton live term births (*≥*37 weeks' gestation) with no evidence of pre-eclampsia (defined according to the International Society for the Study of Hypertension in Pregnancy criteria 2001) or a previous diagnosis of hypertension (*N* = 11,650). All weight and blood pressure measurements, which were taken routinely as part of antenatal care by midwives or obstetricians, were abstracted from obstetric records by six trained research midwives. This resulted in a median of 14 (interquartile range 11 to 16) blood pressure measurements and median of 12 (interquartile range 10 to 14) weight measurements per woman. MAP was calculated as 1/3 × systolic blood pressure + 2/3 × diastolic blood pressure. Confounding variables considered here are maternal height, age, parity, education, smoking and offspring sex. Maternal age and offspring sex were obtained from obstetric records and maternal height, parity, highest educational qualification and smoking status were obtained from questionnaires administered during pregnancy. Smoking status was classified as ‘never’, ‘immediately prepregnancy or first trimester only’, or ‘throughout pregnancy’ for women who continued to smoke after the first trimester. Table 1 shows the characteristics of the women included in the analysis. Linear spline random effects models have previously been fitted to weight measurements in pregnancy, to model changes in weight with gestational age 2011. A similar method was used to fit a linear spline random effects model to MAP (see Section 5).

**Table 1:** Characteristics of the dataset (*N* = 11,650) and the subset with complete data on all covariates (*N* = 9429).

Maternal characteristic	*N* with data	Mean (SD) or %	Mean (SD) or % in complete case
**Height (cm)**	10,278	164.0 (6.69)	164.1 (6.65)
**Age (yrs)**	11,650		
< 20		4.84	3.45
20 − 24		19.30	16.66
25 − 29		38.66	39.56
30 − 34		27.60	29.95
35 +		9.60	10.38
**Parity**	10,830		
Nulliparous		44.56	44.63
Multiparous		55.44	55.37
**Smoking in pregnancy**	10,945		
Never		66.20	68.69
Prepregnancy/first trimester		13.85	13.41
Throughout		19.95	17.90
**Highest educational qualification**	10,492		
CSE/vocational		29.89	28.10
O level		34.51	34.99
A level		22.57	23.35
Degree		13.03	13.55
**Sex of offspring**	11,650		
Male		51.12	50.79
Female		48.88	49.21

## 3 Multilevel spline model with a multivariate response

### 3.1 Multivariate response linear spline random effects model

Multilevel models for repeated measurements have been described by Goldstein 1995 and others. We will first consider a univariate MLM for repeated measurements of a response variable *y*, measured on occasions, *j* = 1, … ,*r*_*k*_ for each individual *k* = 1, … . ,*n*, with two levels: *measurement occasion*_*j*_ within *individual*_*k*_. The response variable is modelled as a linear function of time and the intercept and linear slopes are allowed to vary at the individual level by including individual-level random effects for these parameters as follows:


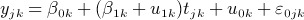


where *y*_*jk*_ is the value of the response variable, *y*, at the *j*^th^ measurement for the *k*^th^ individual, *t*_*jk*_ is the time of the *j*^th^ measurement for the *k*^th^ individual, *u*_0*k*_ and *u*_1*k*_ are the individual level (level-2) residuals for the intercept and slope respectively and the ϵ_0*jk*_s are the measurement occasion level (level-1) residuals. These parameters have the following assumptions:


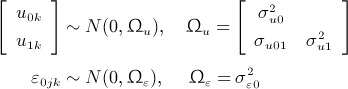


A bivariate response model to investigate linear change in two variables *y*^(*i*)^ for *i* = 1,2 over time has three levels: *responses* ^(*i*)^ within *measurement occasion*_*j*_ within *individual*_*k*_ and thus a model with a random intercept and random slope at the individual level is defined by the following formula and assumptions 1995:


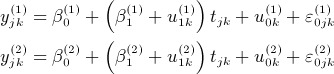



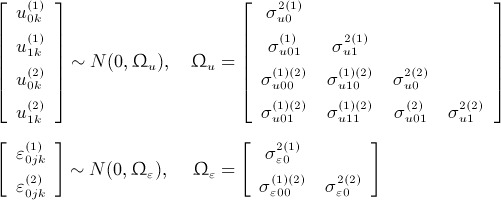


To extend this to a model where time is represented by a set of *m*^(*i*)^ linear splines for response (*i*) where, for *m*^(*i*)^ splines there are *m*^(*i*)^ + 1 time points, 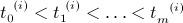 then


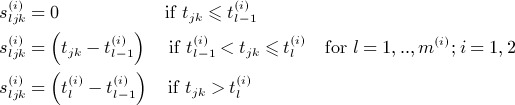


Hence, a linear spline model with a bivariate response has the form


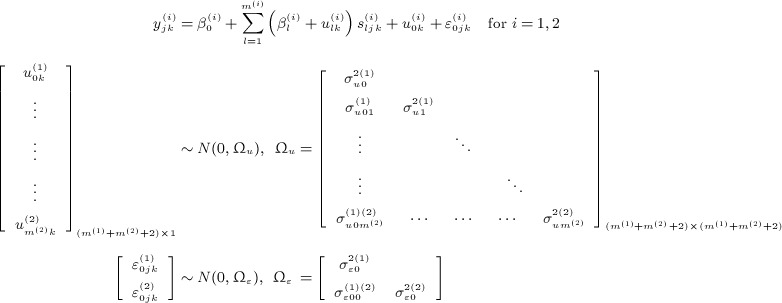


The individual-level random effects give each individual's deviance from the average intercept (*u*_0*k*_) and from the average slope of each of the splines 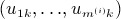 and the individual-level covariance matrix contains the pair wise covariances between each set of these random effects for the intercept and slopes within each of the response variables and between the response variables. For simplicity we have defined a bivariate response model, but this can be extended to more than two response variables. In this case the covariance matrix at the individual level would include all variances and covariances between individual-level residuals and the covariance matrix at the occasion level would include all variances and covariances between occasion-level residuals.

Of note, the model estimated variances and covariances of individual-level random effects, 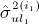, 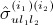, are not the same as the variances and pairwise covariances of the estimated individual-level residuals produced by MLMs, 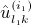, 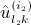, because the residuals are shrunken towards the population mean by varying degrees, reflecting the level of uncertainty with which each individual's residual is estimated 1991. Because the individual-level variance–covariance matrix estimated by the model provides estimates of the population variances and covariances of the random effects this should be used to assess associations between the intercepts and slopes in the population rather than the shrunken residuals.

### 3.2 Testing hypotheses about temporal relationships between response variables

The model defined in Equation (5) allows the individual-level random effects relating to the intercepts and each of the slopes of the response variables in different time periods to be correlated with each other. Specific hypotheses about time periods in which changes in each of the variables are associated may be applied by setting the covariances between the corresponding pairs of the random effects to be zero, thus simplifying the model. The hypotheses may be tested by comparing the simplified models with the full unrestricted model. The fit of the models may be compared by using a likelihood ratio test, with the difference in degrees of freedom between the models being the number of constrained covariances.

For simplicity of explanation, in the following example hypotheses it will be assumed that the same splines are used for both response variables; however, these definitions can easily be adapted to take into account time periods with different endpoints.

### Hypothesis

#### Changes in each of the variables are only associated in the same or adjacent time periods

This hypothesis can be tested by fitting a model with covariances between individual-level random effects relating to change in nonadjacent time periods to be zero:


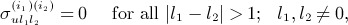


and comparing the fit of this model with the full model with no constraints. The hypothesis can be used to test whether change in one variable in one time period has long-term associations with change in the other variable, or whether it is only associated with change in the immediately subsequent time period.

### Hypothesis

#### Changes in variable 1 are associated with subsequent changes in variable 2, but changes in variable 2 are not associated with subsequent changes in variable 1

For this hypothesis, in the restricted model, all covariances between random effects relating to change in variable 2 and random effects relating to change in variable 1 in a later time period are set to zero:


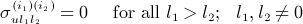


This can be used to test for evidence that changes in variable 1 precede changes in variable 2, rather than changes in variable 2 preceding change in variable 1.

### Hypothesis

#### There is a lag time between change in one variable and change in another

To test this hypothesis, in the restricted model the covariance between random effects relating to change in one variable in a particular time period and random effects relating to change in the other variable in subsequent time periods is set to zero for any time period where it is hypothesised that there may be a lag, but covariances with random effects relating to change in time periods after the hypothesised lag time remain unconstrained


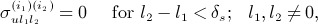


where *δ*_*s*_ is the number of splines/time periods that make up the hypothesised lag time.

The choice of hypotheses to be tested should be guided by the research question, which the analysis is designed to answer, and prior knowledge about the plausible relationships between the variables studied.

### 3.3 Deriving regression coefficients from the random effects variance–covariance matrix

From the covariance matrix of the individual-level random effects relating to baseline values of the response variable or rates of change in these variables in each time period described by the splines it is possible to derive regression coefficients to describe the associations between baseline values and rates of change in each of the variables in different time periods. The regression coefficient for the change in random effect 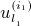 associated with a unit change in random effect 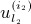 is given by 2001


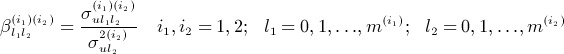


This formula can be extended to give regression coefficients for the relationship between two variables when adjusting for others. Fisher described how to adjust regression coefficients for additional variables 1925 and expressions for regression coefficients when adjusting for up to four covariates are given in the online supplemental material 1.

In the more general case, to regress random effect 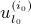 on *p* independent random effect variables: 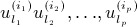, let ***β*** = (*β*_1_,*β*_2_, … ,*β*_*p*_) ′ be the *p* × 1 vector of regression coefficients relating to 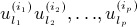, respectively, adjusting for each of the other variables. Also, let 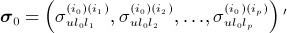 be the *p* × 1 vector of covariances between 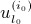 and each of the independent variables and


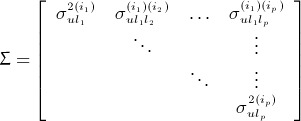


be the *p* × *p* matrix of variances and pairwise covariances of the independent variables. Then Σ***β*** = ***σ***_0_, meaning that the mutually adjusted regressions can be found by solving the equation:


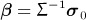


### 3.4 Standard errors of regression coefficients derived from the random effects variance–covariance matrix

We consider a number of methods for calculating the standard errors of the regression coefficients derived from the random effects variance–covariance matrix and compare the performance of these methods on the example dataset in Section 5. The first method estimates the standard errors as if we had sampled *n* observations of the individual-level random effects from an underlying population (where *n* is the number of individuals in our MLM), whereas Methods 2 and 3 estimate the standard errors by treating the regression coefficients as nonlinear combinations of the estimates of the variances and covariances of the random effects, but do this in different ways.

## Method

If we treat our estimates of the variances and covariances of the random effects as if they were obtained from a sample of the individual-level random effects, then the standard errors of the regression coefficients can also be derived using formulae described by Fisher 1925. For the general case with *p* independent variables, where ***β***, Σ and ***σ***_0_ are as in (17) above, let ***d*** be the *p* × 1 vector containing the diagonal elements of the inverse of Σ, 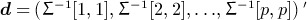. Then the *p* × 1 vector of standard errors, ***SE***_*β*_ = (*SE*(*β*_1_),*SE*(*β*_2_), … ,*SE*(*β*_*p*_)) ′ is given by


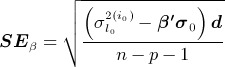


## Method

The second approach is to use the delta method to approximate the standard errors 26,27. This can be implemented in STATA (StataCorp LP, College Station, Texas) using the command nlcom and by explicitly defining the formulae for the regression coefficients from the estimated variances and covariances of random effects (see supporting information for formulae)‡. We note that some programs estimate variances and covariances on transformed scales 2011, and implementations of the delta method are also available in R (The R Foundation for Statistical Computing, Vienna, Austria) 2011. This method is practical when adjusting for up to three or even four variables; however, because there is no convenient expression for the inverse of a square matrix of order greater than four, a method that solves the inverse of Σ numerically (see Method 3) is more appropriate when adjusting for more variables.

## Method

The third approach is to generate a large number, *q*,of realisations of a (*m*^(1)^ + *m*^(2)^ + 2) × 1 vector, which we shall call 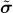, containing all of the variances and covariances of the random effects from the multivariate multilevel spline model (as described in Equation (5)), 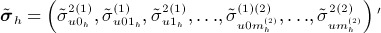 for *h* = 1,2, … ,*q*, using a multivariate normal distribution, 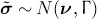. The mean vector, ***ν***, of the multivariate normal distribution is the (*m*^(1)^ + *m*^(2)^ + 2) × 1 vector of estimates of the variances and covariances of the random effects from the MLM, 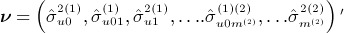, and the 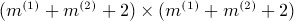 variance–covariance matrix, Γ, is the matrix of the variances and covariances of these estimates, 
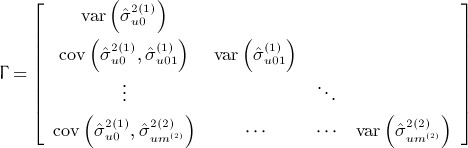
.

After producing *q* realisations of 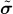, the coefficients relating to the regression of one of the random effects on a subset *p* of the random effects from the MLM are derived for each of the realisations of 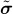 separately. The *h*^th^ realisations of Σ, 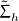, and ***σ***_0_, 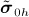, defined in (7) are formed from the estimates in 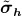 and the equation 
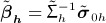
 is solved for each of the *q* realisations. The overall estimate of the vector of regression coefficients 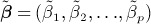 is produced by taking the mean of the *q* realisations, 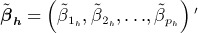, of each of these regression coefficients, 
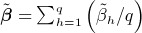
. 95% confidence intervals for each of the elements of 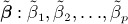 are then produced by using the 2.5^th^ and 97.5^th^ percentiles of their distributions over the *q* realisations as the lower and upper limits, respectively.

We have uploaded STATA commands that implement Methods 2 and 3, named reffadjust4nlcom and reffadjustsim, respectively, to the Statistical Software Components archive 2012.

## 4 Comparison with a structural equation model

Structural equation models contain a measurement equation and a structural equation 31,32. In the measurement equation, each of the measurements 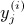 loads on the latent variables 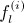 where *l* = 0,1, … ,*m*^(*i*)^. The intercept term for variable *i* is 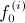, and the loadings for each of the measurements 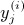 on this latent variable are 1. The other *m*^(*i*)^ factors represent the slopes for each of the periods defined by the splines. Using the same *m*^(*i*)^ + 1 time points, 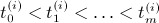 as above, the measurement equation is


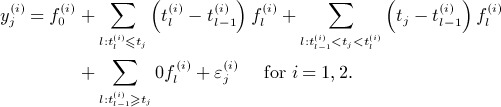


Any rescaling of this equation proportional to these loadings can also be used. To see that this is equivalent to Equation (5), an extra subscript representing individuals, *k*, is added and 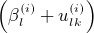 is substituted for each of the 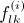. This gives


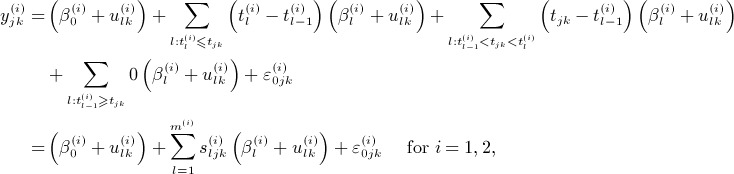


where 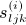 is defined as in Equation (4).

The structural equation of the SEM defines the mutual relationships between the factors. It can be shown that the regression coefficient for the univariate relationship between two factors, 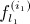 regressed on 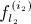 is equivalent to that between two MLM random effects 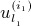 and 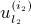 (given in (6)) by substituting 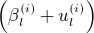 for each of the 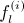 as follows:


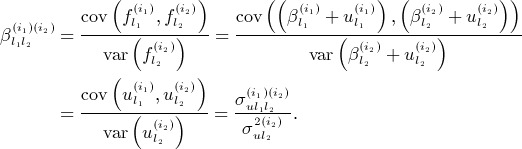


Adjusted regression coefficients for the relationships between factors and their standard errors can be shown to be equivalent to those between random effects in a similar way.

**Figure 1:**
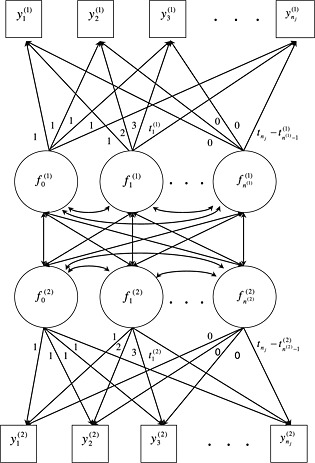
Path diagram for bivariate response spline model in structural equation format. Path diagram assumes that measurements 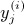 occur at equally spaced time points.

The MLM described in Equation (5) is equivalent to the SEM shown in the path diagram in Figure 1. There have been a number of papers demonstrating the equivalence of linear MLMs and SEMs when each individual has the same number of measurements occurring at the same time points meaning that the data are balanced 31–33. It has also been shown that SEMs can be estimated equivalently to MLMs when data are unbalanced, by treating the design as balanced with missing data and using full information maximum likelihood estimation 2003. However, nonlinear MLMs and generalized linear MLMs do not always have equivalent parametrisations within the SEM framework 7,33. For our weight and MAP example, where individuals have different numbers of measurements and these were taken at different times for each individual, this can be modelled in an SEM by including all times when measurements were taken on any individual (rounded to whole week periods) and treating a particular individual's measurements as missing for any time points on which they were not measured.

## 5 Example: bivariate model of weight and MAP change in pregnancy

**Figure 2:**
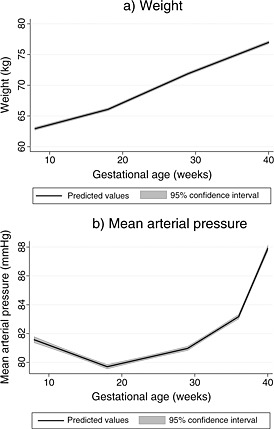
Average trajectories of (a) weight and (b) mean arterial pressure across pregnancy predicted by univariate multilevel linear spline models (*N* = 11,650).

Where there was more than one blood pressure or weight measurement in a 2-week period, one was selected at random for the analysis to prevent any individual from having too high an influence on the models. This left a median of 10 and range of 1 to 18 measurements per woman for analysis. Linear spline models were fitted to weight and MAP separately to describe the shapes of the average trajectories of weight and MAP with gestational age 11,21. Full details of this process are provided in the online supporting information. The model that minimised the differences between predicted and actual values over pregnancy for weight had two knot points, at 18 and 28 weeks' gestation, and for MAP had three knot points, at 18, 30 and 36 weeks' gestation. For simplicity, because the first two knot point locations were similar for weight and MAP and altering the position of the second knot point by 1 week had little effect on the fit of these models, we have used knot points of 18 and 29 weeks for weight and 18, 29 and 36 weeks for MAP. Baseline was set at 8 weeks' gestation for both weight and MAP because there were few measurements prior to this and model predictions earlier in pregnancy than this may be unreliable. Gestational age was rounded to the nearest whole week to make the MLM comparable with the SEM. The formulae for the separate multilevel spline models for weight and MAP, without any confounders included in models were


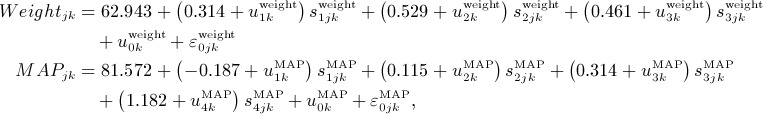

where 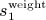 is the first gestational age spline for weight, up to 18 weeks' gestation, 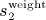 is the second spline, from 18 − 29 weeks, and 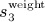 is the third spline, from 29 weeks onwards. For MAP 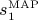 is the first gestational age spline, up to 18 weeks' gestation, 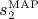 is the second spline, from 18 − 29 weeks, 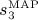 is the third spline, from 29 − 36 weeks and 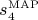 is the fourth spline, from 36 weeks onwards. The shape of the average weight and MAP trajectories across gestation predicted from these models are shown in Figure 2 , and Figure S1 shows example predicted trajectories of weight and MAP for five individual women.

We combined both of these separate spline models into one bivariate spline model, with two responses: weight and MAP. The distributions of weight and MAP at 8 weeks and rates of change in weight and MAP in each period of pregnancy predicted by this unadjusted bivariate model are shown in Table SI. We then included the potential confounders: maternal height (continuous variable); age ( < 20, 20 − 24, 25 − 29, 30 − 34, 35 + years); parity (nulliparous, multiparous); smoking (never, prepregnancy/first trimester, throughout pregnancy); education (CSE/vocational, O level, A level, degree) and offspring sex (male, female). Maternal height was centred about the mean (164 cm) and the reference categories for the other variables were: age 25 − 29 years, nulliparous, never smoked, O level qualification and male offspring. We completed analyses using STATA version 11.2 and MLWIN version 2.24 with runmlwin 2011 for the MLMs and MPLUS version 5 for the SEMs.

The level 2 (individual-level) covariance matrix and correlations between individual-level random effects for this model, with all covariances between random effects freely estimated, are shown in Table II. We will call this MLM Model 1. We then considered several restricted models and tested different hypotheses about the relationships between changes in weight and MAP by comparing these restricted models to the full model (MLM Model 1). MLM Model 2 was constrained so that changes in weight and MAP could only be correlated with weight and MAP change in the same and adjacent periods, but weight and MAP at baseline were allowed to correlate with changes in weight and MAP in all periods. In MLM Model 3 MAP change was not allowed to correlate with weight change in subsequent periods of pregnancy, but weight change was allowed to correlate with later MAP changes, and in MLM Model 4 weight change was not allowed to correlate with MAP change in subsequent periods of pregnancy, but MAP change was allowed to correlate with later weight changes. These models (MLM Models 3 and 4) were used to test whether changes in weight precede changes in MAP or vice versa. In MLM Model 5, MAP change up to 18 weeks was not allowed to correlate with weight at baseline or weight changes in any period, to test whether baseline weight and changes in weight were only associated with increases in MAP after 18 weeks, but not associated with MAP change prior to 18 weeks, when it is decreasing and less likely to be influenced by HDP. Table III shows the deviances of each of the models tested. The fits of the constrained models were all much poorer than the full model (MLM Model 1) so this was selected as the model that best represented the relationships in the data and all of the alternative hypotheses were rejected in favour of this unrestricted model.

**Table II:** Covariances (standard errors) (upper right triangle and diagonal) and correlations (lower left triangle) between individual-level random effects in the full multilevel model (MLM Model 1; *N* = 9429).

	Weight at 8 weeks	Weight change 8 − 18 weeks	Weight change 18 − 29 weeks	Weight change 29 weeks onwards	MAP at 8 weeks	MAP change 8 − 18 weeks	MAP change 18 − 29 weeks	MAP change 29 − 36 weeks	36 weeks onwards
Weight at 8 weeks	119.611	− 0.585	− 0.317	0.044	25.574	0.387	− 0.530	0.196	− 0.435
	(1.786)	(0.037)	(0.025)	(0.027)	(1.244)	(0.142)	(0.093)	(0.143)	(0.276)
									
Weight change	− 0.24	0.050	0.011	0.005	− 0.113	0.001	0.012	− 0.012	0.003
8 − 18 weeks		(0.001)	(0.001)	(0.001)	(0.031)	(0.004)	(0.003)	(0.004)	(0.008)
									
Weight change	− 0.16	0.28	0.033	0.018	− 0.124	0.006	0.011	0.000	0.006
18 − 29 weeks			(0.001)	(0.001)	(0.023)	(0.003)	(0.002)	(0.003)	(0.005)
									
Weight change	0.02	0.10	0.47	0.044	− 0.018	0.006	0.006	0.021	0.027
29 weeks onwards				(0.001)	(0.026)	(0.003)	(0.002)	(0.003)	(0.006)
									
MAP at 8 weeks	0.39	− 0.08	− 0.11	− 0.01	36.579	− 1.071	− 0.278	0.127	− 0.176
					(1.444)	(0.144)	(0.087)	(0.135)	(0.268)
									
MAP change	0.08	0.01	0.07	0.07	− 0.42	0.180	− 0.035	− 0.029	0.015
8 − 18 weeks						(0.020)	(0.011)	(0.016)	(0.031)
									
MAP change	− 0.14	0.16	0.18	0.08	− 0.14	− 0.24	0.113	− 0.042	− 0.012
18 − 29 weeks							(0.010)	(0.012)	(0.020)
									
MAP change	0.03	− 0.09	0.00	0.17	0.04	− 0.12	− 0.21	0.352	− 0.108
29 − 36 weeks								(0.023)	(0.034)
									
MAP change	− 0.03	0.01	0.03	0.11	− 0.03	0.03	− 0.03	− 0.16	1.314
36 weeks onwards									(0.077)

* Model is adjusted for maternal height, age, parity, smoking in pregnancy, education and offspring sex.

**Table III:** Model fit comparisons for full and restricted multilevel models adjusted for confounders (*N* = 9429).

Model	Deviance	Difference in df (compared with model 1)	χ^2^*p*-value
MLM Model 1: Full model	984211.981	—	—
MLM Model 2: Changes in MAP or	984277.574	8	< 0.001
weight are only associated in adjacent			
periods of gestation			
MLM Model 3: Weight change precedes	984241.507	3	< 0.001
MAP change			
MLM Model 4: MAP change precedes	984235.532	5	< 0.001
weight change			
MLM Model 5: Weight at baseline and	984228.987	4	0.002
changes in weight are only associated			
with MAP changes after 18 weeks			

*Note*:

MLM Model 1: Full model with no restrictions.

MLM Model 2: Individual-level random effects for periods of MAP and weight change are correlated only with MAP and weight changes in concurrent and immediately subsequent periods, but not with later periods (although MAP and weight at 8 weeks (intercept) may correlate with MAP and weight changes in any time period).

MLM Model 3: Individual-level random effects for periods of MAP change are not correlated with weight change in later periods of gestation; weight change can correlate with later MAP changes.

MLM Model 4: Individual-level random effects for periods of weight change are not correlated with MAP change in later periods of gestation; MAP change can correlate with later weight changes.

MLM Model 5: Individual-level random effects for weight at 8 weeks and periods of weight change are not correlated with MAP change between 8 and 18 weeks (but may correlate with later MAP changes).

We also fitted an SEM to the data as in Figure 1 with each time point representing a week of pregnancy up to 44 weeks. The earliest week of gestation for which we had data was week 2, producing 43 time points. We included four splines of gestational age for MAP with the same knot points as in the MLM and adjusted for the same set of confounders. All intercept and slope latent variable variances and covariances were freely estimated, but the residual variances for weight in each time period were constrained to be equal, the residual variances for MAP in each time period were constrained to be equal and a common residual covariance between weight and MAP at each time point was also estimated, to make equivalence with MLM Model 1. Table IV shows the covariances and correlations between the intercept and slope latent variables for each of the splines for weight and MAP estimated from the SEM in MPLUS. The covariances and correlations are very similar to the full MLM Model (MLM Model 1). We also compared the estimates of mean weight and MAP at 8 weeks' gestation (baseline) and the mean weight and MAP slopes in each period of gestation between multilevel and structural equation models that were not adjusted for confounders and these were equivalent in the MLM and SEM.

**Table IV:** Covariances (standard errors) (upper right triangle and diagonal) and correlations (lower left triangle) of intercept and slope latent variables in the full SEM (*N* = 9429).

	Weight at 8 weeks	Weight change 8 − 18 weeks	Weight change 18 − 29 weeks	Weight change 29 weeks onwards	MAP at 8 weeks	MAP change 8 − 18 weeks	MAP change 8 − 29 weeks	MAP change 29 − 36 weeks	MAP change 36 weeks onwards
Weight at 8 weeks	119.620	− 0.585	− 0.317	0.044	25.582	0.386	− 0.530	0.197	− 0.436
	(1.788)	(0.037)	(0.025)	(0.027)	(1.232)	(0.141)	(0.092)	(0.143)	(0.271)
Weight change	− 0.24	0.050	0.011	0.005	− 0.114	0.001	0.012	− 0.012	0.002
8 − 18 weeks		(0.001)	(0.001)	(0.001)	(0.032)	(0.004)	(0.003)	(0.004)	(0.008)
									
Weight change	− 0.16	0.27	0.033	0.018	− 0.123	0.005	0.011	0.000	0.006
18 − 29 weeks			(0.001)	(0.001)	(0.023)	(0.003)	(0.002)	(0.003)	(0.005)
									
Weight change	0.02	0.11	0.47	0.044	− 0.018	0.006	0.006	0.021	0.027
29 weeks onwards				(0.001)	(0.026)	(0.003)	(0.002)	(0.003)	(0.006)
									
MAP at 8 weeks	0.39	− 0.08	− 0.11	− 0.01	36.582	− 1.072	− 0.278	0.127	− 0.176
					(1.180)	(0.149)	(0.087)	(0.135)	(0.264)
MAP change	0.08	0.01	0.06	0.07	− 0.42	0.180	− 0.035	− 0.029	0.015
8 − 18 weeks						(0.020)	(0.011)	(0.016)	(0.031)
									
MAP change	− 0.14	0.16	0.18	0.09	− 0.14	− 0.25	0.113	− 0.042	− 0.012
18 − 29 weeks							(0.010)	(0.012)	(0.020)
									
MAP change	0.03	− 0.09	0.00	0.17	0.04	− 0.12	− 0.21	0.352	− 0.108
29 − 36 weeks								(0.023)	(0.034)
									
MAP change	− 0.03	0.01	0.03	0.11	− 0.03	0.03	− 0.03	− 0.16	1.314
36 weeks onwards									(0.081)

* Model is adjusted for maternal height, age, parity, smoking in pregnancy, education and offspring sex.

Table shows the associations of weight at 8 weeks and GWG in each period of pregnancy with MAP changes in the same and subsequent periods of gestation, with and without adjustment for weight and MAP at baseline and earlier changes in these variables. These regression coefficients were derived using the variances and covariances of random effects from MLM Model 1, as set out in Equation (7). The three different methods for producing the standard errors described in Section 3.4 are compared in Table V. We did not use the delta method (Method 2) for regression models that adjusted for more than three variables because this method involved specifying an explicit formula for each of the regression coefficients and these quickly became extremely complex when adjusting for more variables, and impractical to implement. Confidence intervals using Method 1, which assumed the regression coefficients had been obtained from a sample of observations of the individual-level random effects, were much narrower than for Methods 2 and 3, which treated the regression coefficients as nonlinear transformations of the estimates of variances and covariances of the random effects, and these two methods produced very similar confidence intervals.

**Table V:** Mean differences in mean arterial pressure at 8 weeks and average changes in mean arterial pressure in each period associated with a 10 kg increase in weight at 8 weeks or 400 g/week increase in weight gain in the same or earlier periods of gestation, using variances and covariances of the random effects from MLM Model 1 (*N* = 9429).

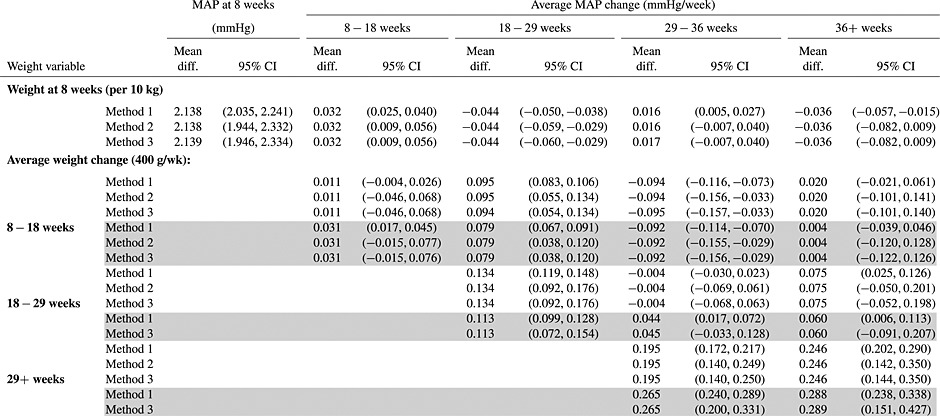

All coefficients are adjusted for maternal height, age, parity, smoking, education and offspring sex (by including these in the bivariate multilevel model); shaded cells are also adjusted for weight and MAP at 8 weeks and weight and MAP changes in periods prior to the exposure period (by including these random effects in the regression).

Method 1: Regression coefficients and standard errors calculated as if we had a sample of individual-level random effects, using Equations (7) and (8).

Method 2: Regression coefficients and standard errors produced using the delta method, implemented by nlcom in Stata.

Method 3: Regression coefficients produced by averaging over 10,000 realisations of the variance-covariance matrix of random effects, with 95% confidence intervals formed from the 2.5^th^ and 97.5^th^ percentiles of the distribution of the regression coefficients over the 10,000 generated matrices.

Table V shows that weight at 8 weeks' gestation was positively associated with MAP at 8 weeks and with MAP change between 8 and 18 weeks, but negatively associated with MAP change between 18 and 29 weeks. GWG between 8 and 18 weeks was not strongly associated with MAP in the same period but was positively associated with MAP change between 18 and 29 weeks and negatively associated with MAP change between 29 and 36 weeks, and these associations remained similar when adjusting for weight and MAP at baseline. GWG between 18 and 29 weeks was positively associated with MAP changes in the same period but not in subsequent periods and GWG from 29 weeks onwards was positively associated with MAP changes between 29 and 36 weeks and from 36 weeks onwards, in models with and without adjustment for weight and MAP at baseline and earlier changes in these variables.

We also considered associations of MAP at 8 weeks and MAP changes with subsequent changes in weight, to examine whether there was evidence that MAP increases may influence greater weight gain for example through increased oedema (Table SII). In models that were unadjusted for baseline weight and earlier GWG and MAP changes, MAP at 8 weeks was negatively associated with GWG in all three periods of gestation and MAP changes between 8 and 18 weeks, and between 18 and 29 weeks were positively associated with GWG in all subsequent periods. However, after adjustment for weight at 8 weeks, there was little evidence that MAP at 8 weeks was associated with changes in weight across pregnancy, except for a weak association with GWG between 18 and 29 weeks. MAP change between 8 and 18 weeks was not associated with GWG between 18 and 29 weeks but was weakly positively associated with GWG from 29 weeks onwards in models adjusted for weight at 8 weeks and GWG and MAP changes in earlier periods of gestation. MAP change between 18 and 29 weeks was not associated with subsequent GWG in fully adjusted models.

## 6 Discussion

We have shown that linear splines for two or more change processes can be used in a multivariate response MLM to investigate associations between rates of change in adjacent periods and to determine whether change in one time period in one variable is associated with change in another variable in the next time period, with time periods defined by the splines. It is also possible to adjust for the value of one or both of the variables at baseline or changes in the variables in earlier time periods.

We compared several methods of deriving standard errors for the regression coefficients for the associations between changes in different time periods and found that standard errors using Method 1, which acted as though we had a sample of individual-level random effects rather than estimates of their variances and covariances, produced confidence intervals that were too narrow and overestimated the evidence against the null hypothesis. The delta method is an appropriate way of estimating the variance of linear and nonlinear transformations of parameter estimates 2003, and incorporated the variance of the estimates of the variances and covariances of random effects in the standard errors of the regression coefficients derived from these. Using percentiles of the distribution of the regression coefficients to combine estimates over 10,000 simulated datasets produced comparable confidence intervals for these regression coefficients to those produced using the delta method. However, it should be noted that these methods (Methods 2 and 3) both assume that the distributions of the variances and covariances of the random effects have a multivariate normal distribution, and so should only be used for large samples where this approximation is justified.

We presented regression coefficients for the associations of pregnancy weight and MAP at baseline and rates of change in these variables with subsequent rates of change in the other variable, both with and without adjustment for weight and MAP at baseline and changes in earlier periods of pregnancy for comparison. This was to assess whether associations between pregnancy weight and MAP change were confounded by earlier changes or baseline values. In general, careful consideration is needed to identify which variables to adjust for to tease out evidence for causal relationships and this decision should be motivated by a particular hypothesis to be tested or by prior knowledge of the relationships between the variables studied.

Previous studies have used covariation in the random effects of two variables in MLMs, either measured at a single point in time 2010 or longitudinally by examining the covariance between random intercept and random slope terms in linear growth models 7,08 to investigate relationships between two variables or rates of change in these variables measured in the same individuals. However, the advantage of using linear splines is that the timings of associations can be identified. For this example analysis we were able to assess whether changes in weight or MAP preceded changes in the other variable, and whether this occurred in all periods of pregnancy or only in early or late pregnancy. This is not possible with a bivariate linear growth model. The linear splines also have the advantage of representing nonlinear patterns of change in an interpretable way, so that the correlations between periods of linear change are more meaningful than correlations between random effects for squared or cubic terms in polynomial models would be. We could also extend the model to include categorical outcomes, provided that the assumption of normally distributed random effects was not violated by including these variables. It is also possible to include different sets of splines for each of the variables if patterns of change in the variables are different.

Cross-lagged SEMs have been used effectively with panel data to demonstrate possible causal relationships between two or more variables measured at several points in time 36,37 and Granger investigated causal relationships between two time series by assessing whether earlier values of each of the variables predicted later observations of the other variable 1969. The synchronous common factor model may be used when a latent variable is thought to influence the two measured processes simultaneously 2001. These models have the flexibility to include prior information by adding constraints and removing unlikely paths from the models. However, the cross-lagged panel design relies on observations being made at the same point in time for each individual and for each measure, and these models are restricted to researcher-determined timing of measurements, rather than time points that may be more biologically meaningful.

Linear MLMs can be equivalently estimated using the SEM framework with both balanced and unbalanced data structures 31–33 but many nonlinear and generalised linear MLMs cannot be equivalently estimated as SEMs 7,33. The MLM approach is a more efficient method of fitting linear models with unbalanced data because within the MLM framework it is not necessary for data to be measured for all individuals at all time points, provided that missingness is at random, whereas in SEMs missing data must be dealt with in some way 2008 such as by full information maximum likelihood estimation. Also, the data management and code become complicated in an SEM when individuals have different numbers of measurements and these occur at different times for each individual, and the models are computationally intensive if there are many possible time points. Furthermore, it is possible to fit a wider range of hierarchical models within the MLM framework 2008. However, a particular advantage to the SEM framework is its high flexibility to specify complex covariance structures, which may not fit into standard MLMs, and the ability to include latent outcome or predictor variables, which are measured by a set of indicator variables 2003. It is also possible to investigate mediation by estimating direct and indirect effects within the SEM framework and obtain tests of overall model fit, which are not available in MLM software 2003. Curran suggested that there was no benefit to using an SEM instead of a standard MLM, but that SEMs could be used to incorporate additional model features that could not be included in an MLM 2003. The multivariate spline MLMs described here could be extended within the SEM framework to produce growth mixture models from the multivariate splines, where patterns of change in two or more variables are used as indicators of latent class membership to extract subgroups of the population with similar change trajectories.

The MLMs and maximum likelihood procedure used in the SEM models to deal with missing data both assume that the data are missing at random, meaning that the missingness in the data can be explained by observed variables 2002. If there is reason to suspect that the missing data mechanism is nonignorable, there are methods available to test the sensitivity of inferences to data being not missing at random for multivariate longitudinal data 2010.

We have demonstrated that these models can be fitted in both MLM and SEM frameworks. The SEM took longer to converge than the MLM, because of the low coverage of the data. All of the women had far fewer weight and MAP observations than the number of time periods used and the SEM treated each time period in which the individual did not have a measurement as a missing value that needed to be estimated using maximum likelihood estimation. However, despite the difficulties with estimation in the SEM, the variance–covariance matrix of the latent intercept and slope factors produced by this model was very similar to that of the individual-level random effects produced by the MLM.

Convergence can also be an issue in MLMs, especially if there are many splines and therefore many variances and covariances of individual level random effects to be estimated. If the model does not converge or calculates negative variances for some of the random effects, it is not possible to derive regression coefficients from these. The choice of the number of splines to use for each variable should depend on the pattern of change in the variable over time, and should be sufficient to achieve an acceptable fit to the observations. It is possible to include a greater number of splines if there is a larger sample size and a high number of measurements per individual, and convergence may be easier if the variables are measured at the same time points for each individual and fewer covariates are included in the model. Difficulties in achieving convergence could be overcome by adding one random effect at a time to the model and reestimating using initial values from the previous model, using fewer splines, or by reducing the accuracy of the timings of the observations, for example rounding gestational age to whole weeks rather than the nearest day, because this reduces the variability in measurement times between individuals. The complexity of the model fitted in our example appeared to be close to the limit of what is possible with this dataset, as in some of our constrained models convergence became difficult.

In summary, we found that GWG up to 18 weeks' gestation was associated with a greater increase in MAP in the next period of pregnancy ( 18 − 29 weeks), but associated with a smaller rise in MAP later on ( 29 − 36 weeks). GWG between 18 and 29 weeks was positively associated with concurrent increases in MAP but not with MAP in later periods, and GWG from 29 weeks onwards was also associated with concurrent MAP. There was some evidence that MAP change was also related to later GWG, with a weak positive association between MAP change up to 18 weeks and GWG from 29 weeks onwards. This method could be applied to many other areas where it is of interest whether changes in one variable precede changes in another, and in what time periods these relationships exist and are strongest. For example it could be used to investigate whether changes in weight are related to changes in respiratory function or bone mass or whether changes in socioeconomic position over time precede changes in a wide range of health and social outcomes.

## References

[b1] Pan H, Goldstein H (1998). Multi-level repeated measures growth modelling using extended spline functions. Statistics in Medicine.

[b2] McCarthy A, Hughes R, Tilling K, Davies D, Davey SG, Ben-Shlomo Y (2007). Birth weight; postnatal, infant, and childhood growth; and obesity in young adulthood: evidence from the Barry Caerphilly growth study. American Journal of Clinical Nutrition.

[b3] Fraser A, Hughes R, McCarthy A, Tilling K, Davies D, Rumley A, Lowe GDO, Davey SG, Ben-Shlomo Y (2008). Early life growth and hemostatic factors. American Journal of Epidemiology.

[b4] Ben-Shlomo Y, McCarthy A, Hughes R, Tilling K, Davies D, Davey SG (2008). Immediate postnatal growth is associated with blood pressure in young adulthood - The Barry Caerphilly growth study. Hypertension.

[b5] Howe LD, Tilling K, Galobardes B, Davey SG, Gunnell D, Lawlor DA (2012). Socioeconomic differences in childhood growth trajectories: at what age do height inequalities emerge?. Journal of Epidemiology and Community Health.

[b6] Howe LD, Tilling K, Galobardes B, Davey SG, Ness AR, Lawlor DA (2010). Socioeconomic disparities in trajectories of adiposity across childhood. International Journal of Pediatric Obesity.

[b7] MacCallum RC, Kim C, Malarkey WB, KiecoltGlaser JK (1997). Studying multivariate change using multilevel models and latent curve models. Multivariate Behavioral Research.

[b8] Suvak MK, Walling SM, Iverson KM, Taft CT, Resick PA (2009). Multilevel regression analyses to investigate the relationship between two variables over time: Examining the longitudinal association between intrusion and avoidance. Journal of Traumatic Stress.

[b9] Pickles A, Croudace T (2010). Latent mixture models for multivariate and longitudinal outcomes. Statistical Methods in Medical Research.

[b10] Fraser A, Tilling K, Macdonald-Wallis C, Sattar N, Brion MJ, Benfield L, Ness A, Deanfield J, Hingorani A, Nelson SM, Davey SG, Lawlor DA (2010). Association of maternal weight gain in pregnancy with offspring obesity and metabolic and vascular traits in childhood. Circulation.

[b11] Macdonald-Wallis C, Tilling K, Fraser A, Nelson SM, Lawlor DA (2011). Established pre-eclampsia risk factors are related to patterns of blood pressure change in normal term pregnancy: Findings from the Avon Longitudinal Study of Parents and Children (ALSPAC). Journal of Hypertension.

[b12] Steegers EA, von DP, Duvekot JJ, Pijnenborg R (2010). Pre-eclampsia. Lancet.

[b13] Lewis G (2007). The, Confidential Enquiry into Maternal and Child Health, CEMACH. Saving Mothers' Lives: Reviewing Maternal Deaths to Make Motherhood Safer-2003-2005. The Seventh Report on Confidential Enquiries into Maternal Deaths in the United Kingdom.

[b14] Duley L (2009). The global impact of pre-eclampsia and eclampsia. Seminars in Perinatology.

[b15] Gaillard R, Steegers EAP, Hofman A, Jaddoe VWV (2011). Associations of maternal obesity with blood pressure and the risks of gestational hypertensive disorders. The Generation R Study. Journal of Hypertension.

[b16] Beyerlein A, Schiessl B, Lack N, von Kries R (2011). Associations of gestational weight loss with birth-related outcome: a retrospective cohort study. British Journal of Obstetrics and Gynaecology.

[b17] Viswanathan M, Siega-Riz AM, Moos MK, Deierlein A, Mumford S, Knaack J, Thieda P, Lux LJ, Lohr KN (2008).

[b18] IOM (Institute of Medicine) and NRC (National Research Council) (2009). Weight Gain During Pregnancy: Reexamining the Guidelines.

[b19] Golding J, Pembrey M, Jones R (2001). ALSPAC-The Avon Longitudinal Study of Parents and Children - I. Study methodology. Paediatric and Perinatal Epidemiology.

[b20] Brown MA, Lindheimer MD, de Swiet M, Van Assche A, Moutquin JM (2001). The classification and diagnosis of the hypertensive disorders of pregnancy: Statement from the International Society for the Study of Hypertension in Pregnancy (ISSHP). Hypertension in Pregnancy.

[b21] Fraser A, Tilling K, Macdonald-Wallis C, Hughes R, Sattar N, Nelson SM, Lawlor DA (2011). Associations of gestational weight gain with maternal body mass index, waist circumference, and blood pressure measured 16 y after pregnancy: The Avon Longitudinal Study of Parents and Children. American Journal of Clinical Nutrition.

[b22] Goldstein H (1995). Multilevel Statistical Models.

[b23] Robinson GK (1991). That BLUP is a good thing: the estimation of random effects. Statistical Science.

[b24] Tilling K, Sterne JAC, Wolfe CDA (2001). Multilevel growth curve models with covariate effects: application to recovery after stroke. Statistics in Medicine.

[b25] Fisher RA (1925). Chapter 5: Tests of significance of means, differences of means, and regression coefficients. Statistical methods for research workers.

[b26] Oehlert GW (1992). A note on the Delta method. American Statistician.

[b27] Greene WH (2003). Econometric Analysis.

[b28] Buis ML (2011). Stata tip 97: Getting at rho's and sigma's. The Stata Journal.

[b29] Jackson CH (2011). Multi-state models for panel data: The msm package for R. Journal of Statistical Software.

[b30] Palmer T, Macdonald-Wallis C http://ideas.repec.org/c/boc/bocode/s457403.html.

[b31] Steele F (2008). Multilevel models for longitudinal data. Journal of the Royal Statisitcal Society: Series A (Statistics in Society).

[b32] Curran PJ (2003). Have multilevel models been structural equation models all along?. Multivariate Behavioral Research.

[b33] Bauer DJ (2003). Estimating multilevel linear models as structural equation models. Journal of Educational and Behavioral Statistics.

[b34] Leckie G, Charlton C

[b35] Van Minh H, Huong D, Wall S, Chuc N, Byass P (2010). Multilevel analysis of covariation in socioeconomic predictors of physical functioning and psychological well-being among older people in rural Vietnam. BMC Geriatrics.

[b36] Burkholder GJ, Harlow LL (2003). An illustration of a longitudinal cross-lagged design for larger structural equation models. Structural Equation Modeling.

[b37] Liu H, Powers DA (2007). Growth curve models for zero-inflated count data: An application to smoking behavior. Structural Equation Modeling.

[b38] Granger CWJ (1969). Investigating causal relations by econometric models and cross-spectral methods. Econometrica.

[b39] Dormann C (2001). Modeling unmeasured third variables in longitudinal studies. Structural Equation Modeling.

[b40] Little R, Rubin D (2002). Statistical Analysis with Missing Data.

[b41] Mahabadi SE, Ganjali M (2010). An index of local sensitivity to non-ignorability for multivariate longitudinal mixed data with potential non-random dropout. Statistics in Medicine.

