# Supplementary material for: Multivariate multilevel spline models for parallel growth processes: application to weight and mean arterial pressure in pregnancy
Source: Stat Med. 2012 Jun 26;31(26):3147–64. doi: 10.1002/sim.5385 (PMC3569877; doi:10.1002/sim.5385)
Supplement: Supplementary file 1 [file sim0031-3147-SD1.pdf]

Supplemental Material I

Corrie Macdonald-Wallis, Debbie A Lawlor, Tom Palmer, Kate Tilling

1 Expressions for regression coefficients adjusting for 2, 3, and 4 covariates

1.1 Two covariates

In a linear model with outcome Y, and covariates X_1, X_2, in a column matrix $\boldsymbol{X} = [1, X_1, X_2]$, we estimate parameters $\boldsymbol{\beta} = [\beta_0, \beta_1, \beta_2]'$. The equation for these parameters is[1]

$$\widehat{\beta} = (\boldsymbol{X}'\boldsymbol{X})^{-1}\boldsymbol{X}'Y \tag{1}$$

From this it can be shown

$$\beta_1 = \frac{\text{var}(X_2)\,\text{cov}(X_1, Y) - \text{cov}(X_1, X_2)\,\text{cov}(X_2, Y)}{\text{var}(X_1)\,\text{var}(X_2) - (\text{cov}(X_1, X_2))^2}$$

$$\beta_2 = \frac{\text{var}(X_1)\,\text{cov}(X_2, Y) - \text{cov}(X_1, X_2)\,\text{cov}(X_1, Y)}{\text{var}(X_1)\,\text{var}(X_2) - (\text{cov}(X_1, X_2))^2}$$

1.2 Three covariates

With three covariates, X_1, X_2 and X_3, the vector $\boldsymbol{X} = [1, X_1, X_2, X_3]$ and we estimate parameters $\boldsymbol{\beta} = [\beta_0, \beta_1, \beta_2, \beta_3]'$ to solve equation (1). Let us define

$$\begin{aligned} D_3 = &\text{var}(X_1)\,\text{var}(X_2)\,\text{var}(X_3) + 2\,\text{cov}(X_1, X_2)\,\text{cov}(X_1, X_3)\,\text{cov}(X_2, X_3) \\ &- \text{var}(X_1)(\text{cov}(X_2, X_3))^2 - \text{var}(X_2)(\text{cov}(X_1, X_3))^2 - \text{var}(X_3)(\text{cov}(X_1, X_2))^2 \end{aligned}$$

Then it can be shown that

$$\beta_1 = \frac{a_{11} \operatorname{cov}(X_1, Y) + a_{12} \operatorname{cov}(X_2, Y) + a_{13} \operatorname{cov}(X_3, Y)}{D_3}$$

$$\beta_2 = \frac{a_{21} \operatorname{cov}(X_1, Y) + a_{22} \operatorname{cov}(X_2, Y) + a_{23} \operatorname{cov}(X_3, Y)}{D_3}$$

$$\beta_3 = \frac{a_{31} \operatorname{cov}(X_1, Y) + a_{32} \operatorname{cov}(X_2, Y) + a_{33} \operatorname{cov}(X_3, Y)}{D_3}$$

where

$$a_{11} = \operatorname{var}(X_2) \operatorname{var}(X_3) - (\operatorname{cov}(X_2, X_3))^2$$

$$a_{12} = \operatorname{cov}(X_1, X_3) \operatorname{cov}(X_2, X_3) - \operatorname{var}(X_3) \operatorname{cov}(X_1, X_2)$$

$$a_{13} = \operatorname{cov}(X_1, X_2) \operatorname{cov}(X_2, X_3) - \operatorname{var}(X_2) \operatorname{cov}(X_1, X_3)$$

$$a_{21} = \operatorname{cov}(X_1, X_3) \operatorname{cov}(X_2, X_3) - \operatorname{var}(X_3) \operatorname{cov}(X_1, X_2)$$

$$a_{22} = \operatorname{var}(X_1) \operatorname{var}(X_3) - (\operatorname{cov}(X_1, X_3))^2$$

$$a_{23} = \operatorname{cov}(X_1, X_2) \operatorname{cov}(X_1, X_3) - \operatorname{var}(X_1) \operatorname{cov}(X_2, X_3)$$

$$a_{31} = \operatorname{cov}(X_1, X_2) \operatorname{cov}(X_2, X_3) - \operatorname{var}(X_2) \operatorname{cov}(X_1, X_3)$$

$$a_{32} = \operatorname{cov}(X_1, X_2) \operatorname{cov}(X_1, X_3) - \operatorname{var}(X_1) \operatorname{cov}(X_2, X_3)$$

$$a_{33} = \operatorname{var}(X_1) \operatorname{var}(X_2) - (\operatorname{cov}(X_1, X_2))^2)$$

1.3 Four covariates

With four covariates, X_1, X_2, X_3 and X_4, the vector $\boldsymbol{X} = [1, X_1, X_2, X_3, X_4]$ and we estimate parameters $\boldsymbol{\beta} = [\beta_0, \beta_1, \beta_2, \beta_3, \beta_4]'$ to solve equation (1). Let us define

$$
\begin{aligned}
D_4 = &\operatorname{var}(X_1)\operatorname{var}(X_2)\operatorname{var}(X_3)\operatorname{var}(X_4) + 2\operatorname{var}(X_1)\operatorname{cov}(X_2,X_3)\operatorname{cov}(X_2,X_4)\operatorname{cov}(X_3,X_4)\\
&+ 2\operatorname{var}(X_2)\operatorname{cov}(X_1,X_3)\operatorname{cov}(X_1,X_4)\operatorname{cov}(X_3,X_4)\\
&+ 2\operatorname{var}(X_3)\operatorname{cov}(X_1,X_2)\operatorname{cov}(X_1,X_4)\operatorname{cov}(X_2,X_4)\\
&+ 2\operatorname{var}(X_4)\operatorname{cov}(X_1,X_2)\operatorname{cov}(X_1,X_3)\operatorname{cov}(X_2,X_3)\\
&+ (\operatorname{cov}(X_1,X_2))^2(\operatorname{cov}(X_3,X_4))^2 + (\operatorname{cov}(X_1,X_3))^2(\operatorname{cov}(X_2,X_4))^2 + (\operatorname{cov}(X_1,X_4))^2(\operatorname{cov}(X_2,X_3))^2\\
&- \operatorname{var}(X_1)\operatorname{var}(X_2)(\operatorname{cov}(X_3,X_4))^2 - \operatorname{var}(X_1)\operatorname{var}(X_3)(\operatorname{cov}(X_2,X_4))^2\\
&- \operatorname{var}(X_1)\operatorname{var}(X_4)(\operatorname{cov}(X_2,X_3))^2 - \operatorname{var}(X_2)\operatorname{var}(X_3)(\operatorname{cov}(X_1,X_4))^2\\
&- \operatorname{var}(X_2)\operatorname{var}(X_4)(\operatorname{cov}(X_1,X_3))^2 - \operatorname{var}(X_3)\operatorname{var}(X_4)(\operatorname{cov}(X_1,X_2))^2\\
&- 2\operatorname{cov}(X_1,X_2)\operatorname{cov}(X_2,X_3)\operatorname{cov}(X_3,X_4)\operatorname{cov}(X_1,X_4)\\
&- 2\operatorname{cov}(X_1,X_2)\operatorname{cov}(X_2,X_4)\operatorname{cov}(X_3,X_4)\operatorname{cov}(X_1,X_3)\\
&- 2\operatorname{cov}(X_1,X_3)\operatorname{cov}(X_2,X_3)\operatorname{cov}(X_2,X_4)\operatorname{cov}(X_1,X_4)
\end{aligned}
$$

Then it can be shown that

$$\beta_1 = \frac{b_{11}\operatorname{cov}(X_1,Y) + b_{12}\operatorname{cov}(X_2,Y) + b_{13}\operatorname{cov}(X_3,Y) + b_{14}\operatorname{cov}(X_4,Y)}{D_4}$$

$$\beta_2 = \frac{b_{21}\operatorname{cov}(X_1,Y) + b_{22}\operatorname{cov}(X_2,Y) + b_{23}\operatorname{cov}(X_3,Y) + b_{24}\operatorname{cov}(X_4,Y)}{D_4}$$

$$\beta_3 = \frac{b_{31}\operatorname{cov}(X_1,Y) + b_{32}\operatorname{cov}(X_2,Y) + b_{33}\operatorname{cov}(X_3,Y) + b_{34}\operatorname{cov}(X_4,Y)}{D_4}$$

$$\beta_4 = \frac{b_{41}\operatorname{cov}(X_1,Y) + b_{42}\operatorname{cov}(X_2,Y) + b_{43}\operatorname{cov}(X_3,Y) + b_{44}\operatorname{cov}(X_4,Y)}{D_4}$$

where

$$
\begin{aligned}
b_{11} = {} & \operatorname{var}(X_2)\operatorname{var}(X_3)\operatorname{var}(X_4) + 2\operatorname{cov}(X_2,X_3)\operatorname{cov}(X_2,X_4)\operatorname{cov}(X_3,X_4) \\
& - \operatorname{var}(X_2)(\operatorname{cov}(X_3,X_4))^2 - \operatorname{var}(X_3)(\operatorname{cov}(X_2,X_4))^2 - \operatorname{var}(X_4)(\operatorname{cov}(X_2,X_3))^2
\end{aligned}
$$

$$
\begin{aligned}
b_{12} = {} & \operatorname{var}(X_3)\operatorname{cov}(X_1,X_4)\operatorname{cov}(X_2,X_4) + \operatorname{var}(X_4)\operatorname{cov}(X_1,X_3)\operatorname{cov}(X_2,X_3) \\
& + \operatorname{cov}(X_1,X_2)(\operatorname{cov}(X_3,X_4))^2 - \operatorname{var}(X_3)\operatorname{var}(X_4)\operatorname{cov}(X_1,X_2) \\
& - \operatorname{cov}(X_1,X_4)\operatorname{cov}(X_2,X_3)\operatorname{cov}(X_3,X_4) - \operatorname{cov}(X_1,X_3)\operatorname{cov}(X_2,X_4)\operatorname{cov}(X_3,X_4)
\end{aligned}
$$

$$
\begin{aligned}
b_{13} = {} & \operatorname{var}(X_2)\operatorname{cov}(X_1,X_4)\operatorname{cov}(X_3,X_4) + \operatorname{var}(X_4)\operatorname{cov}(X_1,X_2)\operatorname{cov}(X_2,X_3) \\
& + \operatorname{cov}(X_1,X_3)(\operatorname{cov}(X_2,X_4))^2 - \operatorname{var}(X_2)\operatorname{var}(X_4)\operatorname{cov}(X_1,X_3) \\
& - \operatorname{cov}(X_1,X_2)\operatorname{cov}(X_2,X_4)\operatorname{cov}(X_3,X_4) - \operatorname{cov}(X_1,X_4)\operatorname{cov}(X_2,X_3)\operatorname{cov}(X_2,X_4)
\end{aligned}
$$

$$
\begin{aligned}
b_{14} = {} & \operatorname{var}(X_2)\operatorname{cov}(X_1,X_3)\operatorname{cov}(X_3,X_4) + \operatorname{var}(X_3)\operatorname{cov}(X_1,X_2)\operatorname{cov}(X_2,X_4) \\
& + \operatorname{cov}(X_1,X_4)(\operatorname{cov}(X_2,X_3))^2 - \operatorname{var}(X_2)\operatorname{var}(X_3)\operatorname{cov}(X_1,X_4) \\
& - \operatorname{cov}(X_1,X_2)\operatorname{cov}(X_2,X_3)\operatorname{cov}(X_3,X_4) - \operatorname{cov}(X_1,X_3)\operatorname{cov}(X_2,X_3)\operatorname{cov}(X_2,X_4)
\end{aligned}
$$

$$
\begin{aligned}
b_{21} = {} & \operatorname{var}(X_3)\operatorname{cov}(X_1,X_4)\operatorname{cov}(X_2,X_4) + \operatorname{var}(X_4)\operatorname{cov}(X_1,X_3)\operatorname{cov}(X_2,X_3) \\
& + \operatorname{cov}(X_1,X_2)(\operatorname{cov}(X_3,X_4))^2 - \operatorname{var}(X_3)\operatorname{var}(X_4)\operatorname{cov}(X_1,X_2) \\
& - \operatorname{cov}(X_1,X_3)\operatorname{cov}(X_2,X_4)\operatorname{cov}(X_3,X_4) - \operatorname{cov}(X_1,X_4)\operatorname{cov}(X_2,X_3)\operatorname{cov}(X_3,X_4)
\end{aligned}
$$

$$
\begin{aligned}
b_{22} = {} & \operatorname{var}(X_1)\operatorname{var}(X_3)\operatorname{var}(X_4) + 2\operatorname{cov}(X_1,X_3)\operatorname{cov}(X_1,X_4)\operatorname{cov}(X_3,X_4) \\
& - \operatorname{var}(X_1)(\operatorname{cov}(X_3,X_4))^2 - \operatorname{var}(X_3)(\operatorname{cov}(X_1,X_4))^2 - \operatorname{var}(X_4)(\operatorname{cov}(X_1,X_3))^2
\end{aligned}
$$

$$
\begin{aligned}
b_{23} = {} & \operatorname{var}(X_1)\operatorname{cov}(X_2,X_4)\operatorname{cov}(X_3,X_4) + \operatorname{var}(X_4)\operatorname{cov}(X_1,X_2)\operatorname{cov}(X_1,X_3) \\
& + \operatorname{cov}(X_2,X_3)(\operatorname{cov}(X_1,X_4))^2 - \operatorname{var}(X_1)\operatorname{var}(X_4)\operatorname{cov}(X_2,X_3) \\
& - \operatorname{cov}(X_1,X_2)\operatorname{cov}(X_1,X_4)\operatorname{cov}(X_3,X_4) - \operatorname{cov}(X_1,X_3)\operatorname{cov}(X_1,X_4)\operatorname{cov}(X_2,X_4)
\end{aligned}
$$

$$
\begin{aligned}
b_{24} = {} & \operatorname{var}(X_1)\operatorname{cov}(X_2,X_3)\operatorname{cov}(X_3,X_4) + \operatorname{var}(X_3)\operatorname{cov}(X_1,X_2)\operatorname{cov}(X_1,X_4) \\
& + \operatorname{cov}(X_2,X_4)(\operatorname{cov}(X_1,X_3))^2 - \operatorname{var}(X_1)\operatorname{var}(X_3)\operatorname{cov}(X_2,X_4) \\
& - \operatorname{cov}(X_1,X_2)\operatorname{cov}(X_1,X_3)\operatorname{cov}(X_3,X_4) - \operatorname{cov}(X_1,X_3)\operatorname{cov}(X_1,X_4)\operatorname{cov}(X_2,X_3)
\end{aligned}
$$

$$
\begin{aligned}
b_{31} = {} & \mathrm{var}(X_2)\,\mathrm{cov}(X_1,X_4)\,\mathrm{cov}(X_3,X_4) + \mathrm{var}(X_4)\,\mathrm{cov}(X_1,X_2)\,\mathrm{cov}(X_2,X_3)\\
& + \mathrm{cov}(X_1,X_3)(\mathrm{cov}(X_2,X_4))^2 - \mathrm{var}(X_2)\,\mathrm{var}(X_4)\,\mathrm{cov}(X_1,X_3)\\
& - \mathrm{cov}(X_1,X_2)\,\mathrm{cov}(X_2,X_4)\,\mathrm{cov}(X_3,X_4) - \mathrm{cov}(X_1,X_4)\,\mathrm{cov}(X_2,X_3)\,\mathrm{cov}(X_2,X_4)
\end{aligned}
$$

$$
\begin{aligned}
b_{32} = {} & \mathrm{var}(X_1)\,\mathrm{cov}(X_2,X_4)\,\mathrm{cov}(X_3,X_4) + \mathrm{var}(X_4)\,\mathrm{cov}(X_1,X_2)\,\mathrm{cov}(X_1,X_3)\\
& + \mathrm{cov}(X_2,X_3)(\mathrm{cov}(X_1,X_4))^2 - \mathrm{var}(X_1)\,\mathrm{var}(X_4)\,\mathrm{cov}(X_2,X_3)\\
& - \mathrm{cov}(X_1,X_3)\,\mathrm{cov}(X_1,X_4)\,\mathrm{cov}(X_2,X_4) - \mathrm{cov}(X_1,X_2)\,\mathrm{cov}(X_1,X_4)\,\mathrm{cov}(X_3,X_4)
\end{aligned}
$$

$$
\begin{aligned}
b_{33} = {} & \mathrm{var}(X_1)\,\mathrm{var}(X_2)\,\mathrm{var}(X_4) + 2\,\mathrm{cov}(X_1,X_2)\,\mathrm{cov}(X_1,X_4)\,\mathrm{cov}(X_2,X_4)\\
& - \mathrm{var}(X_1)(\mathrm{cov}(X_2,X_4))^2 - \mathrm{var}(X_2)(\mathrm{cov}(X_1,X_4))^2 - \mathrm{var}(X_4)(\mathrm{cov}(X_1,X_2))^2
\end{aligned}
$$

$$
\begin{aligned}
b_{34} = {} & \mathrm{var}(X_1)\,\mathrm{cov}(X_2,X_3)\,\mathrm{cov}(X_2,X_4) + \mathrm{var}(X_2)\,\mathrm{cov}(X_1,X_3)\,\mathrm{cov}(X_1,X_4)\\
& + \mathrm{cov}(X_3,X_4)(\mathrm{cov}(X_1,X_2))^2 - \mathrm{var}(X_1)\,\mathrm{var}(X_2)\,\mathrm{cov}(X_3,X_4)\\
& - \mathrm{cov}(X_1,X_2)\,\mathrm{cov}(X_1,X_3)\,\mathrm{cov}(X_2,X_4) - \mathrm{cov}(X_1,X_2)\,\mathrm{cov}(X_1,X_4)\,\mathrm{cov}(X_2,X_3)
\end{aligned}
$$

$$
\begin{aligned}
b_{41} = {} & \mathrm{var}(X_2)\,\mathrm{cov}(X_1,X_3)\,\mathrm{cov}(X_3,X_4) + \mathrm{var}(X_3)\,\mathrm{cov}(X_1,X_2)\,\mathrm{cov}(X_2,X_4)\\
& + \mathrm{cov}(X_1,X_4)(\mathrm{cov}(X_2,X_3))^2 - \mathrm{var}(X_2)\,\mathrm{var}(X_3)\,\mathrm{cov}(X_1,X_4)\\
& - \mathrm{cov}(X_1,X_2)\,\mathrm{cov}(X_2,X_3)\,\mathrm{cov}(X_3,X_4) - \mathrm{cov}(X_1,X_3)\,\mathrm{cov}(X_2,X_3)\,\mathrm{cov}(X_2,X_4)
\end{aligned}
$$

$$
\begin{aligned}
b_{42} = {} & \mathrm{var}(X_1)\,\mathrm{cov}(X_2,X_3)\,\mathrm{cov}(X_3,X_4) + \mathrm{var}(X_3)\,\mathrm{cov}(X_1,X_2)\,\mathrm{cov}(X_1,X_4)\\
& + \mathrm{cov}(X_2,X_4)(\mathrm{cov}(X_1,X_3))^2 - \mathrm{var}(X_1)\,\mathrm{var}(X_3)\,\mathrm{cov}(X_2,X_4)\\
& - \mathrm{cov}(X_1,X_3)\,\mathrm{cov}(X_1,X_4)\,\mathrm{cov}(X_2,X_3) - \mathrm{cov}(X_1,X_2)\,\mathrm{cov}(X_1,X_3)\,\mathrm{cov}(X_3,X_4)
\end{aligned}
$$

$$
\begin{aligned}
b_{43} = {} & \mathrm{var}(X_1)\,\mathrm{cov}(X_2,X_3)\,\mathrm{cov}(X_2,X_4) + \mathrm{var}(X_2)\,\mathrm{cov}(X_1,X_3)\,\mathrm{cov}(X_1,X_4)\\
& + \mathrm{cov}(X_3,X_4)(\mathrm{cov}(X_1,X_2))^2 - \mathrm{var}(X_1)\,\mathrm{var}(X_2)\,\mathrm{cov}(X_3,X_4)\\
& - \mathrm{cov}(X_1,X_2)\,\mathrm{cov}(X_1,X_4)\,\mathrm{cov}(X_2,X_3) - \mathrm{cov}(X_1,X_2)\,\mathrm{cov}(X_1,X_3)\,\mathrm{cov}(X_2,X_4)
\end{aligned}
$$

$$
\begin{aligned}
b_{44} = {} & \mathrm{var}(X_1)\,\mathrm{var}(X_2)\,\mathrm{var}(X_3) + 2\,\mathrm{cov}(X_1,X_2)\,\mathrm{cov}(X_1,X_3)\,\mathrm{cov}(X_2,X_3)\\
& - \mathrm{var}(X_1)(\mathrm{cov}(X_2,X_3))^2 - \mathrm{var}(X_2)(\mathrm{cov}(X_1,X_3))^2 - \mathrm{var}(X_3)(\mathrm{cov}(X_1,X_2))^2
\end{aligned}
$$

1. Fisher RA. 1925. Chapter 5: Tests of significance of means, differences of means, and regression coefficients, Section 29: Regression with several independent variates. In *Statistical Methods for Research Workers.* Oliver and Boyd, Edinburgh.
