# Supplementary material for: Multivariate multilevel spline models for parallel growth processes: application to weight and mean arterial pressure in pregnancy
Source: Stat Med. 2012 Jun 26;31(26):3147–64. doi: 10.1002/sim.5385 (PMC3569877; doi:10.1002/sim.5385)
Supplement: Supplementary file 2 [file sim0031-3147-SD1.doc]

**Supplemental Material 2**

**Description of fitting the univariate linear spline models for weight and MAP**

The approximate position of knot points for the splines was determined by initially fitting fractional polynomial curves to the repeated weight and MAP values across gestation separately. These had orders of up to 2, and all combinations of fractional polynomials using the set of powers (-2, -1, -0.5, 0, 0.5, 1, 2, 3) of gestational age as described by Royston [1] were fitted to the data. The shape of the best-fitting fractional polynomial curve (the curve which maximised the log-likelihood) was used as a guide to the positions of knot points in the spline models. We fitted 2 and 3 knot models and, for those which had the highest log-likelihoods, compared the fit of the model predicted values to the actual values of weight and MAP respectively over the whole course of pregnancy.

Reference List

1. Royston P, Altman DG. Regression Using Fractional Polynomials of Continuous Covariates - Parsimonious Parametric Modeling. *Journal of the Royal Statistical Society: Series C (Applied Statistics)* 1994; **43**(3):429-467.

**Figure Legends**

**Web-Figure 1 Average trajectory of a) weight and b) mean arterial pressure across pregnancy (black line) with predicted trajectories (solid coloured lines) and 95% prediction intervals (dotted coloured lines) for five individual women**

Web-Table Mean and variability of weight and mean arterial pressure parameters from an unadjusted bivariate linear spline multilevel model (N = 11,650)

|  | **Mean** | **Between-individual standard deviation** | **95% reference range** |
| --- | --- | --- | --- |
| **Weight at 8 weeks (kg)**  **Weight change (kg/wk):**  8-18 weeks  18-29 weeks  29+ weeks  **MAP at 8 weeks (mmHg)**  **MAP change (mmHg/wk):**  8-18 weeks  18-29 weeks  29-36 weeks  36+ weeks | 62.94  0.314  0.529  0.462  81.55  -0.185  0.115  0.315  1.176 | 12.02  0.231  0.193  0.220  6.20  0.421  0.337  0.590  1.175 | (39.38, 86.50)  (-0.139, 0.767)  (0.151, 0.907)  (0.030, 0.894)  (69.39, 93.71)  (-1.010, 0.641)  (-0.546, 0.775)  (-0.841, 1.471)  (-1.126, 3.479) |

Web-Table Regression of weight changes in each period on mean arterial pressure at 8 weeks or mean arterial pressure changes in earlier periods of gestation using variances and covariances of the random effects from MLM Model 1 (N=9,429)^1,2^

| **Blood pressure variable** | | **Mean difference in average weight change (400g/week):** | | | | | |
| --- | --- | --- | --- | --- | --- | --- | --- |
|  |  | **8-18 weeks** | | **18-29 weeks** | | **29+ weeks** | |
|  |  | **Mean difference** | **95% CI** | **Mean difference** | **95% CI** | **Mean difference** | **95% CI** |
| **MAP at 8 weeks (mmHg)** | |  |  |  |  |  |  |
|  | Method 1  Method 2  Method 3 | -0.0077  -0.0077  -0.0077 | (-0.0096, -0.0059)  (-0.0119, -0.0035)  (-0.0119, -0.0035) | -0.0084  -0.0084  -0.0085 | (-0.0100, -0.0069)  (-0.0116, -0.0053)  (-0.0116, -0.0054) | -0.0013  -0.0013  -0.0013 | (-0.0030, 0.0005)  (-0.0048, 0.0022)  (-0.0048, 0.0023) |
|  | Method 1  Method 2  Method 3 | 0.0010  0.0010  0.0010 | (-0.0010, 0.0029)  (-0.0037, 0.0056)  (-0.0037, 0.0057) | -0.0045  -0.0045  -0.0045 | (-0.0061, -0.0029)  (-0.0080, -0.0009)  (-0.0081, -0.0009) | -0.0022  -0.0022  -0.0022 | (-0.0041, -0.0003)  (-0.0062, 0.0018)  (-0.0063, 0.0018) |
| **MAP change (mmHg/wk):** | |  |  |  |  |  |  |
| **8-18 weeks** | Method 1  Method 2  Method 3 |  |  | 0.077  0.077  0.077 | (0.055, 0.098)  (0.001, 0.152)  (0.002, 0.158) | 0.090  0.090  0.090 | (0.065, 0.115)  (0.005, 0.174)  (0.007, 0.182) |
|  | Method 1  Method 2  Method 3 |  |  | 0.069  0.069  0.069 | (0.045, 0.092)  (-0.012, 0.149)  (-0.011, 0.154) | 0.090  0.090  0.090 | (0.062, 0.119)  (0.004, 0.176)  (0.005, 0.183) |
| **18-29 weeks** | Method 1  Method 2  Method 3 |  |  |  |  | 0.124  0.124  0.125 | (0.093, 0.155)  (0.037, 0.211)  (0.038, 0.215) |
|  | Method 1  Method 3 |  |  |  |  | 0.035  0.034 | (0.004, 0.065)  (-0.055, 0.123) |

^1^ All coefficients are adjusted for maternal height, age, parity, smoking, education and offspring sex (by including these in the bivariate multilevel model); shaded cells are also adjusted for weight and MAP at 8 weeks, weight and MAP changes in periods prior to the exposure period and weight changes in the exposure period (by including these random effects in the regression)

^2^ Method 1: Regression coefficients and standard errors calculated as if we had a sample of individual-level random effects, using equations (7) and (8).

Method 2: Regression coefficients and standard errors produced using the delta method, implemented by nlcom in Stata

Method 3: Regression coefficients produced by averaging over 10,000 realisations of the variance-covariance matrix of random effects, with 95% confidence intervals formed from the 2.5^th^ and 97.5^th^ percentiles of the distribution of the regression coefficients over the 10,000 generated matrices
